# Natural Sensations Evoked in Distal Extremities Using Surface Electrical Stimulation

**DOI:** 10.2174/1874120701812010001

**Published:** 2018-01-29

**Authors:** Julia P. Slopsema, John M. Boss, Lane A. Heyboer, Carson M. Tobias, Brooke P. Draggoo, Kathleen E. Finn, Payton J. Hoff, Katharine H. Polasek

**Affiliations:** Hope College, Department of Engineering, 223F Vanderwerf 27 Graves Place, Holland, MI 49423, USA.

**Keywords:** Artificial Sensation, Strain hardening, Electrical Stimulation, Referred Sensation, Surface electrodes, Surface Electrical Stimulation, Phantom Limb

## Abstract

**Background:**

Electrical stimulation is increasingly relevant in a variety of medical treatments. In this study, surface electrical stimulation was evaluated as a method to non-invasively target a neural function, specifically natural sensation in the distal limbs.

**Method:**

Electrodes were placed over the median and ulnar nerves at the elbow and the common peroneal and lateral sural cutaneous nerves at the knee. Strength-duration curves for sensation were compared between nerves. The location, modality, and intensity of each sensation were also analyzed. In an effort to evoke natural sensations, several patterned waveforms were evaluated.

**Results:**

Distal sensation was obtained in all but one of the 48 nerves tested in able-bodied subjects and in the two nerves from subjects with an amputation. Increasing the pulse amplitude of the stimulus caused an increase in the area and magnitude of the sensation in a majority of subjects. A low frequency waveform evoked a tapping or tapping-like sensation in 29 out of the 31 able-bodied subjects and a sensation that could be considered natural in two subjects with an amputation. This waveform performed better than other patterned waveforms that had proven effective during implanted extra-neural stimulation.

**Conclusion:**

Surface electrical stimulation has the potential to be a powerful, non-invasive tool for activation of the nervous system. These results suggest that a tapping sensation in the distal extremity can be evoked in most able-bodied individuals and that targeting the nerve trunk from the surface is a valid method to evoke sensation in the phantom limb of individuals with an amputation for short term applications.

## 1 INTRODUCTION

Electrical stimulation is becoming increasingly relevant for a variety of medical applications. For example, it is used to promote plasticity and recovery of voluntary movements in individuals with spinal cord injury [1] and stroke [2, 3]. Treatments involving electrical stimulation are also being proposed for conditions such as diabetes, asthma, hypertension, arthritis, pain, and cancer [4]. A non-invasive method to target specific neural functions could make such treatments more accessible and acceptable to patients.

Significant progress has been made in electrical activation of tactile sensations to restore sensory feedback to individuals with amputated limbs. Such activation requires surgery to place stimulating electrodes in or around a peripheral nerve in the residual limb. Subjects have experienced tapping, touch, vibration, movement, and other sensations in their phantom limb from this implanted stimulation [5-9]. Sensory restoration has been shown to increase subject performance on a variety of tasks from force matching to object detection [10-12]. These invasive methods have shown significant activation of somatosensation but are less attractive for short-term therapeutic applications. Surface electrical stimulation (SES), a non-invasive stimulation method, is already a component of successful devices [13, 14]. The study described here focused on improving the type of somatosensation obtained from SES, with the aim of developing therapies rather than for long-term restoration of sensory feedback.

Surface electrical stimulation is used clinically to improve function after a stroke [2, 15] or spinal cord injury [13, 16] and to treat intractable pain [14]. When the nerve trunk is targeted, muscle contractions and/or sensations can be produced in distal locations based on the innervation pattern of the nerve [17]. For example, stimulation of the median nerve at the elbow produces muscle activation and/or sensation in the hand. Sensations obtained using SES in this way are often described as tingling or prickling (paresthesia) [18]. In individuals with amputated limbs, paresthesia can be evoked in the missing limb while stimulating on the skin of the residual limb and this technique is being investigated as a treatment for phantom limb pain [19-21]. An improvement in the SES technique to produce non-paresthesia sensations may improve the treatment for phantom limb pain and will improve the knowledge of how to use this valuable stimulation tool for targeted activation.

The individual firing patterns for the different types of mechanoreceptors is well known [22-24]. Most receptors produce a rapid discharge of action potentials during an indentation of the finger (touch/pressure). Then slowly-adapting receptors continue to fire at a reduced rate while rapidly-adapting receptors cease firing. This suggests that the standard, constant frequency square pulse train that has been used historically for motor activation is not appropriate when activating the somatosensory system. In addition, patterned waveforms have been suggested as a method to address the fact that the nervous system is not accustomed to receiving constant information [25]. Tan *et al.* found that slight fluctuations in the pulse duration of a square-pulse waveform reduced paresthesia and resulted in more natural sensations and less paresthesia [8]. It is currently not possible to target individual axons or types of axons using SES but varying the stimulation waveform may improve the quality of the evoked sensations and reduce paresthesia.

The goal of this study was to achieve natural sensation using surface electrical stimulation by varying the stimulation parameters and the waveform. Natural sensation was defined as something that could be reproduced visually, such as touching, stroking, or tapping.

## 2 MATERIALS AND METHODS

### 2.1 Experimental Setup

Able-bodied subjects were recruited from the Hope College campus community; subjects with limb amputations were recruited through Mary Free Bed Rehabilitation Hospital. All subjects were between the ages of 18 and 65. The protocol was approved by the Hope College Human Subjects Review Board and all subjects gave written informed consent. Each study session lasted from 45 to 90 minutes and subjects were compensated for their time.

The experimental setup was similar to what has been described previously [18]. After giving consent, subjects were instructed to wash the area where the electrodes would be applied with soap and water and were seated in an upright chair. Subjects sat with their left arm extended on a table or their left knee partially extended on a padded foot rest. Rubbing alcohol was used to clean the skin before applying the electrodes. For median nerve stimulation, electrodes were placed over the biceps tendon with the active electrode placed proximal to the return Fig. (**1a**). For ulnar nerve stimulation, electrodes were placed over the groove between the olecranon of the ulna and the medial epicondyle of the humerus on the back of the elbow (where the ulnar nerve is most superficial, Fig. (**1b**). For stimulation in the lower extremity, the common peroneal nerve was the preferred target and electrodes were placed over the biceps femoris tendon at the knee Fig. (**1c**). In some subjects, reliable activation of the common peroneal nerve was not obtained and electrodes were moved more medially on the back of the knee to activate the lateral sural cutaneous nerve. A photograph was taken of the electrodes to document their location and an elastic band was wrapped around the arm/leg to assist electrode adhesion when necessary. If a band was used, it was removed as necessary to prevent a decrease in circulation to the limb.

Stimulation was supplied *via* voltage-controlled, charge-balanced, biphasic, non-symmetric square pulses. A non-symmetric waveform was used to reduce the probability of activation during the anodic phase of the pulse. Pulses were non-symmetric in that the anodic phase was set to a maximum value of 4 V with a width as needed to balance the charge [26, 27]. Voltage-controlled stimulation was used to decrease the risk of high current density in the case of reduced adhesion from the surface electrodes. The stimulation waveforms were created in MATLAB (2011a) and delivered using a National Instruments USB DAQ (NI USB-6229, Austin, TX) and an isolated biostimulator (Coulbourn Instruments model A13-75, Pittsburg, PA). Adhesive electrodes were cut to 30 mm by 17 mm (ValuTrode, Axelgaard Manufacturing, Fallbrook, CA).

Prior to stimulation, the limb was hidden from view to prevent a disparity between what was felt and seen. Initially, ramping was performed using 0.5s pulse trains at 50 Hz (with 1s in between) that increased in amplitude by 0.5 V at pulse durations of 100 μs and 500 μs. This allowed the subject to become accustomed to the stimulus and determine approximate sensation threshold values. If muscle contraction was observed the electrode position was adjusted until a satisfactory range without motor activation was obtained. Ramping continued until the subject indicated their maximum comfort level. The subject was then instructed to adjust their arm/leg position until maximum distal sensation was achieved while minimizing sensation under the electrodes. Once an acceptable position was found, subjects were asked to refrain from movements for the remainder of the experiment.

### 2.2 Data Collection

#### 2.2.1 Exploratory Trials

**Fig. (2):**
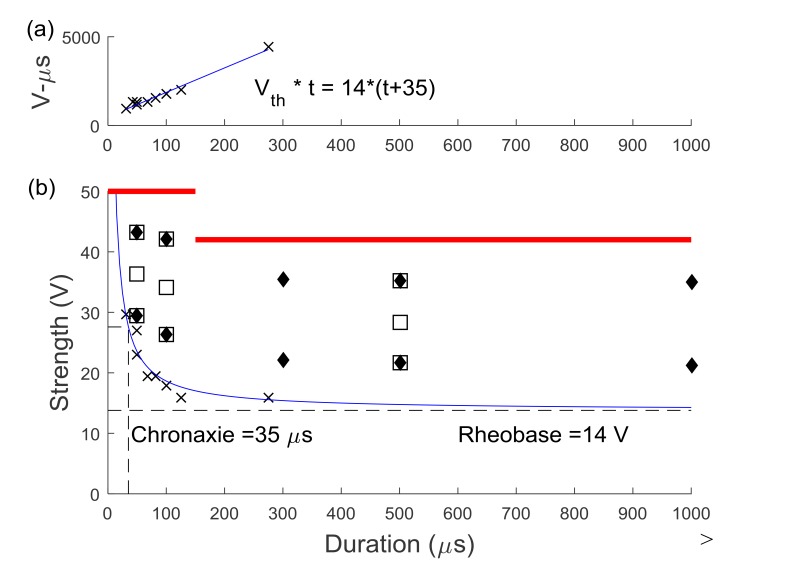
Example strength-duration curve calculation from the ulnar nerve of subject 3. **(A)** The product of pulse amplitude (V_th_) and duration (t) was plotted against pulse duration. This linear relationship was used to calculate rheobase amplitude and chronaxie time using Equation 1. **(B)** Resultant strength-duration curve obtained from Equation 2 (blue) with the same threshold points plotted as ‘x’. The upper red lines are the maximum comfort level from ramping at 100 µs (left portion) and 500 µs (right portion). The filled diamonds represent the 10 pulse amplitude and duration combinations used with the upper extremity nerves and the open squares are the 9 points used for the lower extremity nerves.

An adaptive psychophysical procedure, Parameter Estimation by Sequential Testing (PEST) [28, 29], was used to determine perception threshold values for sensation in the hand or foot using a range of pulse duration and amplitude values. PEST is a method of threshold detection that was developed to obtain a more accurate estimation of sensory data and remove some of the variability and bias that occur when collecting data using subject reporting. The procedure consisted of starting with a subthreshold stimulus and increasing until a hand sensation was reported and then decreasing until the sensation disappeared. This was continued with a decreasing step size until the step size was below either 0.25V or 30µs depending on the parameter being varied. Based on the results of prior studies, efficient calculation of the strength duration curve can be performed using two trials of five threshold values [18]. PEST was used to determine the sensory threshold at two constant pulse durations and three constant pulse amplitudes using 0.5s pulse trains repeating every 1.5s. Two trials to determine threshold were performed in a random order under each of these five conditions and these sensory thresholds were used to calculate the strength-duration curve, the rheobase voltage and the chronaxie time as described by other [18, 30]. Briefly, the product of pulse amplitude (V_th_) and duration (t) was plotted against pulse duration (t). This linear relationship was used to calculate rheobase amplitude and chronaxie time using Equation **1** Fig. (**2a**). The effective parameter space was defined with this strength-duration curve (Equation **2**) as a lower boundary (blue line in Fig. (**2b**) and the maximum comfort level as an upper boundary (red lines in (Fig. **2b**).

$V_{\mathrm{th}}\text{∗}t=V_{\mathrm{Rh}}(t+\tau_{C})$

Vth=VRh(1+τCt)

Ten locations within the effective parameter space (diamonds in Fig. (**2b**) were tested for the median and ulnar nerves with two pulse amplitude levels for each of five pulse durations (50, 100, 300, 500, 1000 µs). Nine locations were tested for the lower extremity (squares in Fig. **2b**), with three amplitude levels for each of three pulse durations (50, 100, 500 µs). The trials were presented in the same random order for each subject and consisted of pulses at 50 Hz sent in repeating 0.5 second trains with 1 second in between. This frequency was chosen because higher frequencies were found to produce painful sensations [9]. For each set of stimulation parameters, subjects were instructed to report the modality, location, and intensity of the sensation in their hand or foot. A list of possible sensation modalities was provided (finger bent, pressing, weight bearing, probing, prickling, tingling, cool, numbness, buzzing, and unnatural) but subjects were also able to use a descriptive term of their choice. To standardize subject reporting, the palmar and dorsal surfaces of the hand were divided into 25 locations based on innervation patterns and expected sensation size Fig. (**3**). The dorsal and plantar surfaces of the foot were divided into 19 locations as seen in Fig. (**3**). Subjects were asked to point out the location of the sensation on a model hand or foot and the pre-defined location(s) that most accurately represented this area was selected in the computer by the experimenter. Intensity was recorded using an open-ended scale to allow relative comparison of strength. These values (modality, location and intensity) were recorded using a Matlab graphical user interface for later analysis.

#### 2.2.2 Additional Waveforms

Prior studies have reported an increase in the quality of the sensation through the use of non-constant or patterned waveforms [8, 25]. This is based on the fact that mechanoreceptor firing rate is rarely constant and varies across receptors [23]. In an effort to obtain more natural sensations, additional trials were performed using a number of patterned stimulation waveforms. The first two waveforms were based on promising results using electrodes implanted around the nerve [8]. All stimulus trains were sent at a pulse amplitude that was 60% of the range between the threshold at which the subject began to feel sensation in their distal limb and the maximum amplitude that the subject considered comfortable. This value was occasionally increased to obtain a more robust sensation using the lower frequency waveforms. Trials were randomized within each set of waveforms. Subjects were asked to state the sensation felt in their hand or foot after each stimulus train using the same sensation list as in the prior trials with the addition of ‘Tapping’. The modality, location and intensity were recorded on data sheets.

Full-scale and small-scale modulation were based on the equations presented by Tan *et al.* [8]. The pulse duration varied based on the base pulse duration (PD_base_), the modulation frequency (f_mod_) and the modulation percentage (M_per_). The period of the pulses (time between the start of two subsequent pulses) remained constant at 0.02s. Equation 3 gives the relationship for the pulse duration, which is also shown in Fig. (**4a**). Actual waveform are shown in Figs. (**4b** and **4c**). For full scale modulation, M_per_ was 1 (100%), PD_base_ was 500 µs, and f_mod_ varied between 1 and 2 Hz. For small-scale modulation, M_per_ was 0.2 (20%), PD_base_ was 500 µs, f_mod_ was 20 Hz. Tan et al used a much lower modulation percentage (about 5%) but this small fluctuation was not found to change the reported sensation during preliminary testing with surface stimulation so a higher percentage was chosen. Within these small scale modulation trials the burst length was varied from 0.05 to 0.5 s and the inter-burst time was varied from 0.05 to 0.9 s.

$\mathrm{Duration}=M_{\mathrm{per}}PD_{\mathrm{base}}\sin(f_{\mathrm{mod}}t)+PD_{\mathrm{base}}$

The final two waveforms involved a low frequency pulse train (1-4 Hz). In one of the waveforms, a pre-burst of 2-5 fast pulses (50 or 100 Hz) preceded the low frequency pulse train. This waveform was based on the firing pattern for slowly adapting mechanoreceptors which fire rapid pulses for a short time due to an indentation and then fire at a slower frequency while the indentation remains constant [23, 31]. The second waveform consisted of the low frequency pulse train (1-4 Hz) without the initial rapid pulses and was originally designed for the subjects to directly compare the two waveforms. The total waveform length was 5 seconds.

### 2.3 Data Analysis

#### 2.3.1 Exploratory Trials

The purpose of the exploratory trials was to determine the effect of varying pulse amplitude and pulse width on modality, location, and intensity of evoked sensation. The percentage of times each sensation was reported for each nerve and each parameter was compared. Changes in intensity and location were assessed by comparing responses when the pulse amplitude was increased at a constant pulse duration.

#### 2.3.2 Additional Waveforms

The purpose of testing different waveforms was to find a waveform that produced realistic sensation with less paresthesia than found with the previously tested 50 Hz constant pulse train. For each nerve and waveform type, the occurrence of each sensation was quantified using the percentage of trials where the sensation was reported. Some similar sensations were grouped together, such as buzzing, tingling, prickling, and numbness (paresthesia). A McNemar’s Test was used to compare the different conditions and comparisons were considered significant if the family error rate was less than 0.05.

## 3 RESULTS

Discernable sensation in the hand or foot was obtained in 49 out of the 50 nerves from 36 different able-bodied subjects and two subjects with an amputated limb. The sensation type varied from tingling to tapping and was consistent with sensory innervation maps [17]. An approximately equal number of subjects of each gender participated, ranging in age from 18 to 47 (average age was 23). Of the subjects with an amputation, one received a transradial amputation following an accident 1.5 years prior to testing. The other received a transtibial amputation due to congenital arteriovenous malformation almost 2 years prior to testing.

### 3.1 Exploratory Trials

Twenty-three able-bodied subjects participated in this study for a total of 10 trials on each nerve, with some subjects participating on more than one nerve. Subjects received intermittent stimulation for an average of 13 minutes to determine the strength-duration curves and an average of 8 minutes for the different amplitude/duration pairs.

#### 3.1.1 Strength-Duration Curves

The strength-duration curves used to define the lower bound of the effective parameter space are shown in Fig. (**5**). There was no significant difference between the rheobase (p=0.58) but there was a difference in the chronaxie time (p=0.03) due to the higher chronaxie in the lower extremity nerves. When compared to previously published data [18], there was no difference between the rheobase and chronaxie values for the median nerve (p=0.06) and ulnar nerve (p=0.12 for rheobase, p=0.45 for chronaxie). The lower extremity curves had more variability than the upper extremity and this was probably due to the fact that the protocol was changed half-way to force a threshold point at a higher pulse duration (500 µs). When the ANOVA was repeated using the final five subjects on each nerve there was no statistical difference (p=0.58). The first curves from the first five subjects are plotted in gray in Fig. (**5c**). The less accurate method could result in a lower rheobase which would lead to a higher chronaxie time since the chronaxie time is the duration threshold at twice the rheobase voltage. This possibly resulted in an underestimated threshold for the first 5 subjects, but since the minimum stimulation was at 25% of the range, foot sensation at this lowest level was reported in all but trial with one subject.

#### 3.1.2 Sensation Modality

All subjects reported sensation in the foot or hand before muscle activation. This sensation was most often described as paresthesia (buzzing, tingling, prickling or numbness), and at least one of these descriptions was used by each subject. Just under half of subjects (30% from median nerve; 33% from ulnar nerve; and 64% from the lower extremity) reported more natural sensations, such as probing, tapping, or pressing at some point during the trials. In the subjects with an amputation, sensations such as pressure, toe bending, and pulsing were reported but paresthesia was once again reported most often.

#### 3.1.3 Sensation Location

At the lowest amplitude of stimulation for each pulse width (25% of the range), most upper-extremity subjects reported sensation in one or two of the pre-defined locations (median of 2; mean of 3; see blue bars in Fig. (**6a**). The number of locations ranged from 0 (no sensation felt) to 9, from UE subject 11 who reported sensation throughout the median nerve innervation area at the higher pulse durations. When stimulating at 75% of the range, the median number of locations increased to 4 with a mean of 3.9 (orange bars in Fig. (**7a**). Out of the 13 subjects who reported sensation in only one of the predefined locations, the most common locations were in the base of the palm (8 total subjects) and 5 subjects across both nerves reported sensation in only one finger tip. All reported locations from 25% and 75% of the range are shown in Figs. (**7a** and **7c**) for median and ulnar nerve respectively. As the stimulus was increased, the number of reports of finger-tip-only sensation decreased as the number of reports of whole finger sensation increased. At 75% of the range, 80% of subjects reported sensation in the entire ulnar nerve innervation area while only 10% reported the entire median nerve area.

**Fig. (7):**
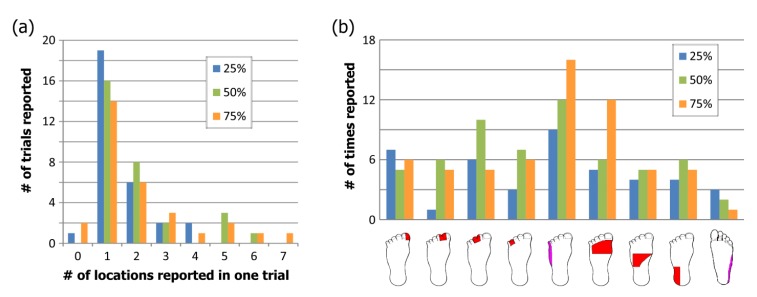
Lower Extremity sensation location. (**a**) The number of predefined locations where sensation was felt during lower extremity stimulation. (**b**) The number of times each location was reported in the 30 trials for each stimulation level. Multiple locations were reported in some trials so the total will be more than 30. The locations that arose from stimulating the lateral sural cutaneous nerve are depicted in purple.

In the foot, just over half of subjects felt sensation in the toes at the lowest amplitude while the rest felt it along the lateral side of the foot. These two disparate locations are likely due to activation of the common peroneal or lateral sural cutaneous nerve in different subjects. The median number of predefined locations where sensation was reported was 1 for all three stimulation levels while the mean increased from 1.5 to 1.97 to 2.07 Fig. (**7a**). Since the predefined locations on the dorsal surface of the foot were large, an increase in sensation size may not have resulted in sensation in more than one predefined location. As the stimulation level increased, there were more reports of sensation on the top of the foot and along the lateral side (Fig. **7b**).

As the pulse amplitude was increased, 76% of the trials on the median nerve had the same or greater area (expansion-Fig. (**8a**), 2% produced a smaller location (reduction) while the rest exhibited some loss in activation from the original area with the addition of new areas (relocation). Relocation varied from a slight loss of the original area to a complete change in the location of the sensation Fig. (**8b**). For the ulnar nerve, expansion occurred in 78% of trials and relocation was found in 16%. In the lower extremity, relocation was more common than in the upper extremity and found in 37% of trials while expansion occurred in 53%. This increase in relocation percentage for the lower extremity was likely due to the fact that the electrodes were near both the lateral sural cutaneous and common peroneal nerves and slight movements of the subject may have shifted the activation from one nerve to the other.

For the subject with an upper limb amputation, sensations were located near the center of the palm. Increasing stimulation did not change this location significantly. However, throughout the course of the stimulation, the subject reported warmth in the phantom limb, where previously the phantom had always been cold. This warmth persisted for several hours after testing concluded but no actual temperature of the limb was recorded. For the subject with a lower limb amputation, sensation started at the base of the big toe and expanded to involve all toes as the amplitude was increased. At higher levels of stimulation, a feeling of cramping in the toes was reported, similar to what was felt by this subject due to a touch on the top of the foot immediately prior to the amputation.

#### 3.1.4 Sensation Magnitude

As the pulse amplitude was increased from 25% of the range of comfortable sensation to 75% of the range, the average normalized sensation magnitude increased significantly for both upper extremity nerves (p<<0.01). All three levels (25%, 50% and 75% of the range) were significantly different in the lower extremity (p<0.05, Tukey-Kramer method). When comparing individual subjects and increasing from 25% of the range to 75%, the magnitude was found to be higher or the same in 92% of pulse amplitude values from all three nerves. In the lower extremity, where there were three pulse amplitude levels at each pulse duration, the magnitude increased in 52 out of 60 comparisons between successive stimulation levels (87%). Similar results were seen in the subjects with amputations with all trial on the median nerve and half of the trial on the common peroneal nerve resulting in increased sensation magnitude. These results suggest that the magnitude of distal sensation can be at least crudely modulated using surface electrical stimulation.

### 3.2 Additional Waveforms

Ten subjects were recruited for each upper extremity nerve and 11 for the lower extremity. All four waveforms described in the method section were performed on the upper extremity nerves while only the low-frequency train and the full scale modulation were tested on the lower extremity nerves. Visually reproducible sensations such as tapping or pressing were reported in all but three nerves in the upper extremity and five in the lower extremity. All but two subjects who did not report tapping or pressing did report pulsing which, although not as natural, was a non-painful, non-paresthesia sensation. One subject (UE9) described the sensations from median nerve stimulation as cool or that the finger was bent. One (UE7) used a range of terms including pulsing, tapping, jerking, pulling, heartbeat and weight bearing. Most subjects reported a similar sensation across all waveforms with varying degrees of paresthesias.

The trials that produced the most non-painful, non-paresthesia sensations used a constant low frequency pulse-train Fig. (**9**). The low frequency pulse train preceded by a burst of high frequency pulses (LF w/pre-burst) resulted in the next highest number of these natural responses but had a significantly higher amount of paresthesia reported (p=0.000015, McNemar’s test). Trials involving the pre-burst also resulted in more reports of ‘uncomfortable’ than the other trials. No reduction in paresthesia was found using full-scale or small-scale modulation, even though these techniques had produced favorable results when using implanted nerve-based stimulation [8].

There was a difference in the reported sensations between the two upper extremity nerves. On the median nerve, there was a drastic decrease in the amount of paresthesia reported with the low frequency waveform compared to waveforms which included the pre-burst (p=0.00024, McNemar’s Test). In the ulnar nerve, little difference was seen in the amount of paresthesias between these two waveforms (p=0.14). This variation in response may be due to the different compositions of somatosensory fibers within the nerves.

In the two subjects with an amputation, only the low frequency waveform was tested. The upper extremity subject reported a pulsing near the center of the phantom palm. The lower extremity subject reported a bending and cramping in the phantom toes. This was an interesting result since cramping of the toes was a common response to touch for this subject immediately prior to amputation. Both subjects reported paresthesia as well.

## 4 DISCUSSION

The purpose of this study was to investigate the types of sensation that could be obtained using surface electrical stimulation with the aim being to obtain natural (non-paresthesia) sensations. Distal sensation was obtained in all but one of the 50 nerves tested. Strength-duration curves were generated for a subset of the subjects and they corresponded well with prior data. Increasing the pulse amplitude of the stimulus caused an increase in the area of the sensation as well as the magnitude of the sensation in a majority of subjects. Buzzing and tingling were the most common sensations reported with all but one waveform type. The waveform that produced the most natural sensations was a low frequency pulse train at 1 to 4 Hz. This waveform performed better than a similar waveform that included a pre-burst as well as other patterned waveforms that had proven effective during implanted extraneural stimulation.

Two subjects with an amputation participated in portions of this study and sensation in their phantom limbs was evoked through stimulation of the proximal nerve trunk. Initially, the lower extremity subject only reported sensation in the intact limb. As ramping stimulation was repeated, the phantom foot “grew back” and sensation was reported in the phantom toes for the remainder of the trial. Neither subject directly reported ‘tapping’ when using the low frequency waveform. The lower extremity subject felt sensations that occurred when the foot was touched immediately prior to it being amputated (cramping/bending of toes) while the upper extremity subject reported pulsing that varied little across trials. In addition, the upper extremity subject reported a relaxing in the portion of the hand innervated by the median nerve while the fourth and fifth fingers stiffened. Finally, both subjects reported short term changes in sensations in their phantom following this trial. The upper extremity subject had sustained warmth in the residual limb and phantom hand while the lower extremity subject had more awareness of the phantom foot for several hours following testing. This is consistent with what has been found by others [9] and was not painful. These are interesting anecdotal reports that need to be investigated further.

### 4.1 Sensation Modality

Paresthesia was reported in a majority of trials, which is consistent with previous work, from both implanted and surface stimulation [9, 18]. When stimulating directly on the nerve, Tan *et al.* found that patterned stimulation improved the quality of the sensations, nearly eliminating paresthesia [8]. In contrast, similar patterned waveforms from surface electrical stimulation did not reduce paresthesia. Tan reported a very small window where the sensation would change from pressure, to pressure with tingle and then to only tingling. In early experiments, attempts were made to locate this window using surface stimulation with no success. The waveforms tested here were found to be the most pleasant during preliminary experiments using surface stimulation and were based on those used by Tan *et al.*

The pre-burst waveform was chosen to mimic the firing of slowly adapting Merkel cells and Ruffini endings to evoke pressure sensations [23, 24, 31]. Instead, it appeared that the stimulation was interpreted as coming from rapidly adapting receptors which are responsible for vibration and tapping sensations. Rapidly adapting (RA) units have been previously reported to evoke tapping when activated individually using intraneural microstimulation [32].

There was a difference in the response to the low-frequency waveform between the median and ulnar nerves. Paresthesia significantly decreased in the median nerve when using a constant, low frequency waveform, but a similar decrease was not seen in the ulnar nerve. The ulnar nerve innervates a smaller area of glabrous skin than the median nerve and RA units make up 43% of sensory units in the glabrous skin [33]. Similarly, the ulnar nerve at the elbow has a lower percentage of cutaneous fibers that innervate the hand (66% for median versus 35% for ulnar) [34]. Therefore, if low-frequency activation of the RA axon seems to be interpreted as tapping, it is possible that the lower number of RA units in the ulnar nerve can explain the lower-quality sensation (higher amount of paresthesia) reported from ulnar nerve stimulation. Even with the higher percentage of paresthesia, all subjects reported a tapping-like sensation at least once during ulnar nerve stimulation.

Pressure or pressing were rarely reported from any of the waveforms. This is a key sensation for sensory feedback systems since prosthetic users need to know how things are pressing on their prostheses. This surface stimulation method was not intended to be used for long term sensory feedback. The goal of this study was to evoke any visually reproducible sensation rather than provide a range of sensations for sensory restoration. The tapping reported by most of the subjects meets this goal.

All subjects were provided with a list of sensation descriptions that may have skewed their responses. However, both natural and unnatural sensations were included on the list in an attempt to not bias responses to our desired outcome. Having the list seemed to help subject get started on describing the sensation and many did end up choosing ‘other’ and reported sensations such as ‘heartbeat’, throbbing’ and ‘vibrating’. Tapping was added as a choice (in the middle of the list) for the Additional Waveform trials since it was most commonly reported during preliminary testing. The sensation options list was a limitation to the study but it was designed to provide options without guidance.

### 4.2 Sensation Location

Increasing the stimulation amplitude was expected to increase the area of nerve that was activated and did result in sensation reported in more location in most trials. When the reported area did not increase, it sometimes changed location and occasionally decreased. These findings were likely due to slight changes in electrode position relative to the nerve that occur when subjects shift their body. In testing the lower extremity, the electrodes were placed near where the lateral sural cutaneous nerve branches off of the common peroneal. Part of the lateral sural cutaneous nerve joins with a branch from the tibial nerve to form the sural nerve. A branch of the sural nerve innervates the side of the foot, while branches from the common peroneal nerve innervate the top of the foot and toes. Both areas of activation were reported by some subjects. Small variations in the electrode location would make it possible to activate either or both of these locations on the foot, leading to the higher variability in sensation location reported in the lower extremity testing.

To make a viable clinical system, an electrode array is currently being developed to allow for adjustment of electrode voltages and resulting nerve activation. This will provide a simple method for the user to adjust sensation location in the distal extremity in response to unwanted changes such as those due to movement.

### 4.3 Sensation Magnitude

In nine of the 16 cases where the sensation magnitude decreased, it only decreased by a reported magnitude of 1. Since the magnitudes were self-reported on an open ended scale and trials were presented in a random order, these sensations could easily be the result of human reporting error. Other situations that resulted in a decrease in reported magnitude were: (1) a different modality was felt between the two trials in question; or (2) a very strong sensation was felt in the trial immediately preceding the 75% amplitude level. Three out of the 16 cases did not fit into any of these categories (out of 160 total comparisons).

Graczyk *et al.* reported smooth psychometric functions when modulating pulse duration or pulse frequency found by asking the subject to choose which of a pair of stimuli had a higher intensity [35]. Their methods removed the limitations that come from using a verbal scale for magnitude reporting. Dhillon et al used a magnitude rating scheme similar to what was performed in the present study and obtained quite varied responses from subjects even with the same stimulation parameters [7]. This suggests that the variability in magnitude results may be due to variability in human reporting more than variability in surface electrical stimulation.

## CONCLUSION

Surface electrical stimulation has the potential to be a powerful, non-invasive tool for the activation of the nervous system. This study reports a technique that evoked tapping or a tapping-like sensation in the distal extremity in 29 out of the 31 nerves tested in able-bodied subjects. Two subjects with amputations also reported sensations in their phantom limb that could be considered natural, even though not specifically tapping. The ability to activate small portions of the hand or foot while stimulating from the surface, proximal to the target extremity, is an example of the level of specificity of nerve activation that can be accomplished non-invasively. Future work will focus on increasing the reliability of surface electrical stimulation to make this technique a clinically viable therapeutic option.

## Figures and Tables

**Fig. (1):**
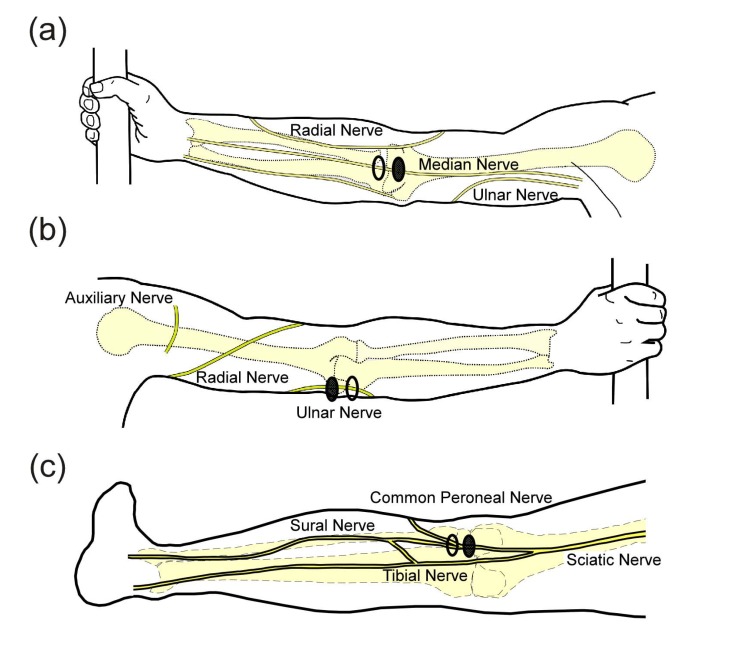
Electrode location for targeting the median (**a**), ulnar (**b**) and common peroneal (**c**) nerves. Filled circles are active electrodes and open circles are return electrodes.

**Fig. (3):**
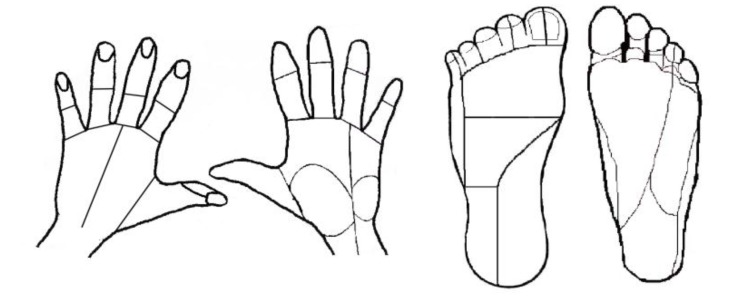
Predefined sensation locations for the left hand and foot. There were 25 locations in the hand and 19 locations in the foot.

**Fig. (4):**
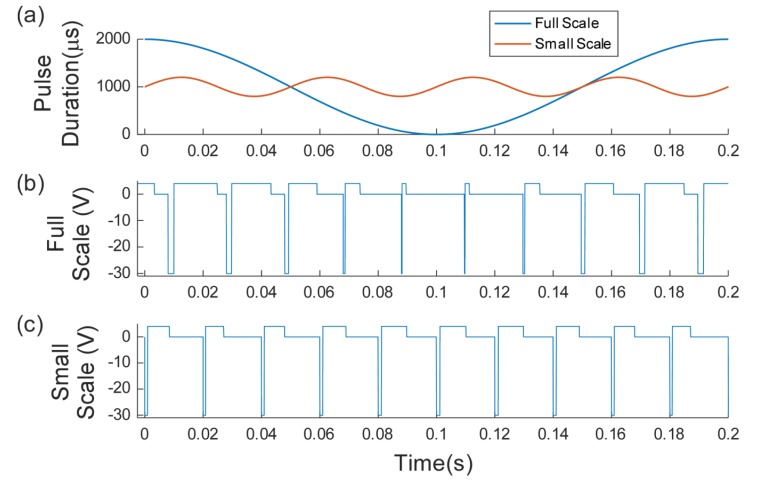
The patterned waveforms utilizing non-symmetric, charge balanced pulse trains at 30 V (maximum recharge voltage of 4 V). (**a**) Description of how the pulse duration was modulated for a base duration of 1000 µs: Full scale modulation (blue) at a modulation frequency of 5 Hz and a modulation percentage of 100%; Small scale modulation (red) at a modulation frequency of 20 Hz and a modulation percentage of 20%. These are slightly different than the experimental values for demonstration purposes. (**b**) Resultant full-scale modulation waveform where the shortest pulse durations occur around 0.1 s. (**c**) Resultant small-scale modulation waveform has more subtle changes as the pulse duration modulates between 800 and 1200 µs.

**Fig. (5):**
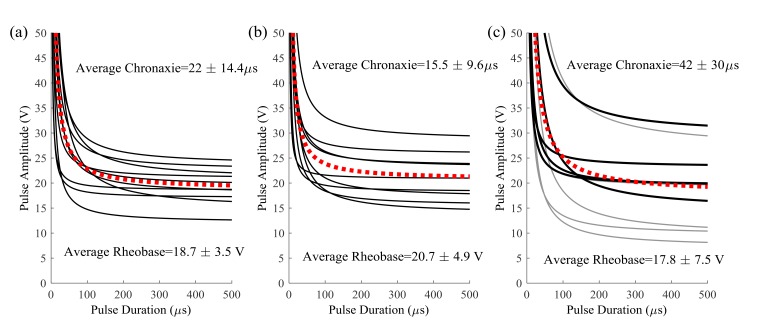
Strength duration curves from each nerve with the average plotted in the thick red dotted line. (**a**) Median nerve; (**b**) Ulnar nerve; (**c**) Lower Extremity with first 5 subjects before the protocol change plotted in gray.

**Fig. (6):**
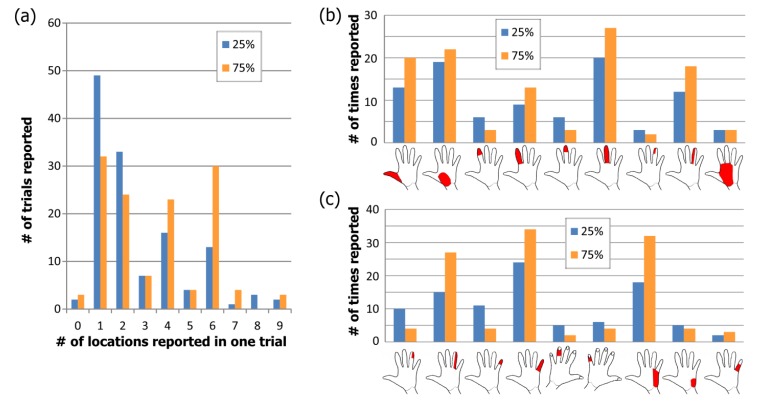
Upper Extremity sensation location. (**a**) The number of predefined locations where sensation was felt during median and ulnar nerve stimulation. (**b & c**) The number of times each location was reported in the 50 trials for each stimulation level for the median (**b**) and ulnar (**c**) nerves. Multiple locations were reported in some trials so the total will be more than 50.

**Fig. (8):**
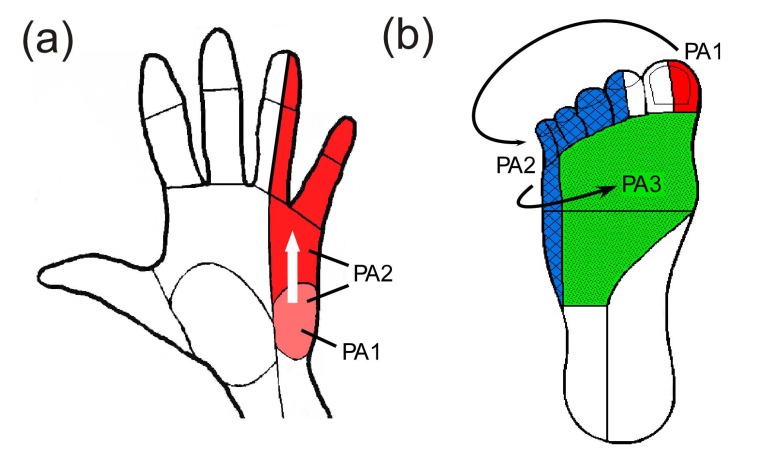
The effect of increasing pulse amplitude. (**a**) Example of expansion with activation of a greater area with increasing pulse amplitude, while maintaining activation in the original area. This result was from Subject 13 on the ulnar nerve stimulating at 300µs. (**b**) Example of relocation with activation of equal or greater area with differences in location. This result was from Subject LE10 stimulating at 100µs. The labels indicate the order in which locations were felt with increasing pulse amplitude (PA).

**Fig. (9):**
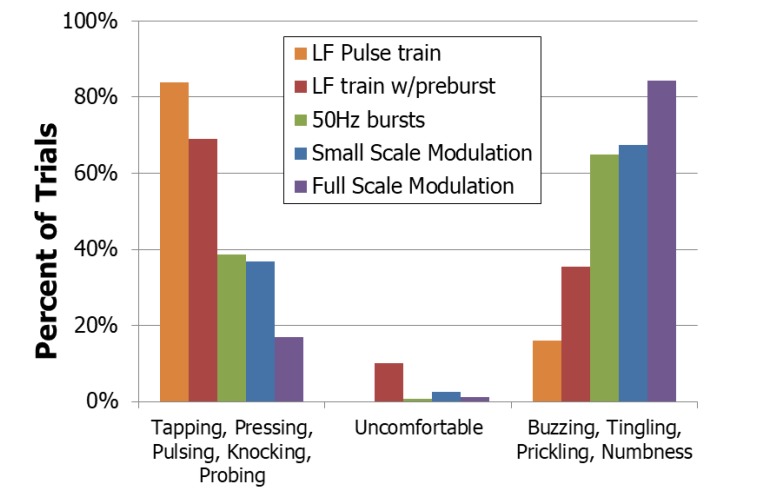
Reported sensations across all trials on all nerves. The low frequency pulse train resulted in the most non-paresthesia sensations with minimal discomfort. Some totals are over 100% since some subjects reported more than one descriptor per trial. Sensations that were reported fewer than three times are not included in this figure.

## LIST OF ABBREVIATIONS

PEST: Parameter Estimation by Sequential Testing
RA: Rapidly Adapting
SES: Surface Electrical Stimulation
